# Prices for veterinary care of dogs, cats and horses in selected countries in Europe

**DOI:** 10.3389/fvets.2024.1403483

**Published:** 2024-07-18

**Authors:** Agneta Egenvall, Odd V. Höglund, Ruben Hoffman, Paul S. Valle, Pia Haubro Andersen, Cecilia Lönnell, Anna Byström, Brenda N. Bonnett

**Affiliations:** ^1^Agneta Egenvall, Odd Höglund, Department of Clinical Sciences, Faculty of Veterinary Medicine and Animal Husbandry, Swedish University of Agricultural Sciences, Uppsala, Sweden; ^2^Ruben Hoffman, Department of Economics, Faculty of Natural Resources and Agricultural Sciences, Swedish University of Agricultural Sciences, Uppsala, Sweden; ^3^Paul S. Valle, Tornes i R, Norway; ^4^Pia Haubro Andersen, Department of Anatomy, Physiology and Biochemistry (AFB); Division of Anatomy and Physiology, Swedish University of Agricultural Sciences, Uppsala, Sweden; ^5^Cecilia Lönnell, Tequi, Stockholm, Sweden; ^6^Anna Byström, Department of Applied Animal Science and Welfare, Swedish University of Agricultural Sciences, Uppsala, Sweden; ^7^Brenda N Bonnett, B Bonnett Consulting, Georgian Bluffs, ON, Canada

**Keywords:** animal insurance, corporate, inflation, veterinarian, veterinary care prices, animal health economics, price transparency, consumer

## Abstract

**Introduction:**

In recent years, prices for veterinary care have received considerable attention in mainstream media, yet scientific literature has not delved into actual figures. This study aims to elucidate veterinary care costs for dogs, cats, and horses across five countries [Sweden (SE), Norway (NO), Denmark (DK), United Kingdom (UK), and Ireland (IR, with limited data)] through web searches.

**Methods:**

Utilising online business directories, we located URLs featuring veterinary care prices in autumn 2022, and repeated tri-monthly five times. Vetpris.se (VP), a price comparison site for SE, NO, and DK, emerged from the search. Additionally, we sought to compare price data from veterinary clinics (ranging from animal hospitals to small private clinics) using a similar approach to VP. We targeted elective procedures (e.g., gonadectomy, GDY) and common procedures (e.g., pyometra surgery in dogs).

**Results:**

Comparing data from the same clinics’ websites and from VP within extraction from autumn 2022 to winter 2023/2024, median prices for dog and cat GDY were largely consistent. By October 2023, median prices for male cat GDY ranged from €72 (SE) to €152 (DK), and €130 (SE) to €269 (NO) for females; for dog GDY from €390 (SE) to €438 (NO) for males, and €461 (UK) to €803 (NO) for females. Across sources, median prices for cat and dog GDY varied from a decrease of 1% to an increase of 31% over a year for procedures with at least 10 clinics per extraction. Equine GDY (per sedation and local analgaesia) in SE saw a 64% increase by year-end, with a median price of €492. Emergency surgeries during regular-hours (e.g., pyometra and caesarean section) in SE were approximately €2,300 at the last extraction, marking a 27% increase for pyometra surgery during regular-hours and 15% after-hours compared to the previous year. Variability existed within and across countries and diagnoses/procedures.

**Discussion:**

Cross-validation suggested VP generally provided reliable information, though data points for emergencies were limited. Our web searching tool necessitated extensive manual verification, indicating room for further development. We recommend enhancing price transparency for animal owners to become better informed about the cost of veterinary care and be able to make informed choices.

## Introduction

Traditionally, veterinarians at individual clinics and hospitals have been responsible for setting prices for veterinary visits and procedures. Over the last decade in Sweden (SE), Norway (NO), and Denmark (DK), the veterinary sector has witnessed some increased price transparency. Clinics often display prices for preventive procedures such as gonadectomy (GDY), vaccinations, and euthanasia, typically on their websites. Nevertheless, prices for other types of care are seldom openly available beforehand and are usually disclosed only when a treatment strategy is proposed. The need for enhanced price transparency across the veterinary care sector was underscored in a recent Swedish Government Official Report (1) and also addressed by competition regulators in the UK (2).

In SE, articles in the public press indicate a significant increase in the prices of veterinary care for pets and horses in recent years, sparking intense public debate (3, 4). It has been suggested that one contributing factor to these higher costs is the entry of private equity firms into the market, leading to considerable market consolidation. This trend began in SE in 2011, with two private equity firms holding a 65% market share by the end of 2012 (5). Between 2013 and 2016, the net turnover of privately owned Swedish veterinary care practices increased by 38%, indicating a correlation between market concentration and rising prices (6), the recent intense debate suggesting further increases (3, 4). Similarly, the veterinary care markets in the UK (7), the US (8), and, albeit at a slower pace, Ireland (9), have experienced increased consolidation with the entry of private equity firms, sparking public debate (10–12). In NO and DK, there have been fewer public reactions to the prices of veterinary care, despite the established presence of private equity firms. The issues of rising prices, market consolidation, and reduced accessibility to appropriate veterinary care for owners have been widely discussed in several countries, including the US and Canada (7, 13, 14).

In SE, insurance coverage for companion animals’ veterinary care has traditionally been common. Recent figures from 2023 indicate that in SE 83% of dogs and 69% of cats were insured (15). In NO, 27% of all pets were insured in 2023 (16), while in the UK, 11% of dogs and 32% of cats (in 2020) were insured (17). In the US, insurance coverage is considerably lower, with only 5% of pets being insured in 2023 (18–20). While it has been argued that higher levels of pet insurance could make veterinary services more affordable (21, 22), it has also been suggested that the high insurance coverage has contributed to increasing veterinary care prices in SE (1, 14, 23). Recently, a group of Swedish insurance companies has expressed concerns that rising veterinary prices may restrict dog ownership to wealthy individuals (4). Some pet insurance products in the UK have been removed from the market in 2023–2024, suggesting that insurance companies can have problems settling veterinary care reimbursements (24).

Research indicates that veterinarians frequently encounter distressed animal owners unable to afford veterinary care fees (21). The significant and escalating expense of veterinary care has been recognised as a potential threat to animal welfare, the well-being of animal owners, and the psychological health of veterinary healthcare staff (13, 25–27). In North America, there is a push for providing a ‘Spectrum of Care’ as a means to enhance accessibility and affordability of veterinary care, along with additional training for veterinarians to provide such services (28), and in the UK ‘Contextualisation of Care’ promotes the idea that care does not always need to be gold standard (25).

Despite these concerns and the observed price increases for veterinary care, along with calls for improved price transparency, there is a lack of compiled data on prices for different types of procedures. Limited attention has been given in scientific literature to quantifying expenses associated with veterinary care for pets and horses.

The primary aim of this study was to describe veterinary care prices in five countries for dogs, cats, and horses by gathering information from veterinary clinics, hospitals, and private practitioners, collectively referred to as veterinary clinics. The focus was on both routine procedures (e.g., GDY) and selected common emergency procedures (e.g., pyometra surgery in dogs) over a 12-month period. Prices were collected directly from veterinary clinics and from a price comparison website, ‘Vetpris’ (VP) (29). In addition to clinic website prices, VP collects client-submitted receipts as evidence of actual prices paid for selected and typical veterinary diagnoses and procedures and standardises those to enable comparisons between clinics. Additional aims were to cross-validate information from VP with that found directly on the internet and to assess the potential of web searching in creating a source of comparative pricing.

## Materials and methods

An ethical permit was not considered necessary for this type of study according to Swedish law. However, it was possible that secondary personal identifiers could be found in the raw data (e.g., if an URL was made up from a veterinarian’s name), therefore it was handled according to routines to preserve confidentiality at The Swedish University of Agricultural Sciences, with the protocol dictating that only two of the authors had access to the raw data. Key parts of the code used to handle the dataset resulting from the procedures below are found in Supplementary Data Sheet 1. All data are presented in summarised format, without clinic identifiers.

Searches for veterinary clinics and practicing veterinarians were made in online business directories covering SE, NO, DK, UK and IR and prices were downloaded when found. Further details on techniques for extraction, cross-checking multiple sources, and filtering data are presented (Supplementary Data Sheet 2).

Data were included over a period of 15 months. The first extraction at the VP-site was not complete for all procedures (i.e., for some procedures not all posts were shown, see further below for specifics of how this was handled). Thus, the web search was initiated in autumn 2022 and was repeated five times with approximate tri-monthly intervals. Web extractions were made 2022-08-30, 2022-09-26 (I), 2023-01-02 (II), 2023-04-07, 2023-04-13 (III), 2023-06-29 (IV), 2023-09-30 (V) and 2024-01-02 (VI). Web and VP-extractions were intended to be simultaneous, but for technical reasons the first extraction from VP was done in 2022-08-30 and the third 2023-04-13.

The results were downloaded to an excel file that contained 337,583 rows for all 6 extractions. Data were managed and analysed in SAS (30). Information included time of extraction, clinic identifiers (name, URLs, and numbering identifiers), procedural and diagnostic information in text and number (from VP) formats, as well as price information. Pricing data was further cleaned in some cases, such as adjusting intended figures into uniform formats for further analysis (e.g., omitting non-breaking blanks or to produce mean prices when prices were given as a range). Prices in national currencies were converted into euros (€), using the conversion rates at the time of extraction (31).

Using geo-coordinates (provided from the search) the locations of the clinics (those in the csv-file) were mapped (32). Clinic URLs were scrutinised for association with a chain of clinics. Affiliations (Supplementary Data 1, sheet links- used for mapping) were retrieved using regular expressions and distinguished in mapping.

The dataset was found to include a wide range of procedures. However, a subset of procedures for which price information was more frequently available (i.e., more observations) was chosen for further analysis. Examples of commonly found prophylactic procedures include GDY of male cats, various vaccinations, euthanasia of dogs of a certain weight, and dental cleaning. Finally, the following procedures, representing both elective procedures and procedures performed because of medical problems, were finally selected:

1. Gonadectomy of male and female cats, of male and female dogs and of male horses (for horses during sedation and local analgaesia), representing elective procedures.
2. Pyometra and caesarean surgery of dogs and cats, representing surgical emergency procedures because of medical problems, both during regular-hours and after-hours.
3. Stifle surgery in dogs, including tibial plateau levelling osteotomy (TPLO), tibial tuberosity advancement (TTA) and extracapsular lateral suture stabilisation surgery, representing surgical procedures because of medical problems.

The freely available VP website (29) is designed so animal owners in need of veterinary care can see from a map which clinics perform various procedures, opening hours, if the clinics are on call or are currently open, and if there is price information from these clinics at the VP website. The platform primarily focuses on dogs, cats, and horses, with the current language set to Swedish and currency in SEK. The data encompasses information from SE, NO, and DK. The VP site displays prices for elective procedures sourced from the internet, as well as prices for various diagnoses and procedures related to the treatment or investigation of acutely or chronically ill patients. Price information from clinic websites is continuously monitored and updated when changes occur (some websites are instead checked monthly). For prices related to procedures and diseases not found online, information is mainly obtained from animal owners who submit de-identified receipts to VP but also sometimes through direct communication with clinics. Receipts are solicited through frequent requests on social media, and a dedicated platform on the VP website allows users to submit receipts. All prices on the VP site are standardised using specific templates. For instance, the template for the surgical removal of a tumour on the outer eyelid in a 30 kg dog includes a consultation fee, veterinary fee, surgery under general anaesthesia, and disposable materials (33). The horse GDY template covers either a 30 km travel fee or a consultation fee, veterinary fee, analgaesia, and routine open GDY (involving the opening of the tunica vaginalis) (34). VP personnel review all submitted receipts to compile standardised prices. Veterinary clinics are encouraged to manage and update their own prices, labelled as ‘clinic prices’ when entered or modified. In the first extraction conducted in the autumn of 2022 only the 20 lowest prices for receipt-based problems could be extracted from the VP website, but since January 2023 all prices are shown. Hence the mean and median prices for some receipt-based procedures prior to 2023 are expected to be lower than if all prices (available at VP within their system) had been included.

### Analysis

For web data prices from the selected list were targeted and tabulated by country, species, and extraction. Price data from the web were retrieved from the diagnosis/procedure field, using regular expressions. To ensure that an outtake was correct, data were iteratively scrutinised, including the text in the diagnosis/procedure field and price outliers, and at times checked against the clinical website for further information. For example, very low or high prices were eliminated based on manually verified outliers (SAS code for male cat GDY can be found in Supplementary Data Sheet 1).

Prices for all diagnoses/procedures from VP (VP-prices) have been tabulated by country, species, and extraction. Price data from VP were reported as found, without any data cleaning (except for when evident outliers were found, see results).

For each procedure only one observation per data source (web, VP), clinic and point in time was used (i.e., duplicates were removed). Especially for dog GDY there were often different prices for dogs of different weights, which occurred both for VP and on clinic websites. In such cases a mean price was calculated. Further, any consultation fee was typically included in GDY prices for cats and dogs (what is included for each procedure is often but not always explained on the clinic homepage). Package prices including GDY, vaccinations and micro-chipping could also often be found on clinic websites. In such cases only the entries without these additions were kept.

Most results are presented in Euro, including between-country comparisons. However, original currency values were also analysed. Further, purchasing power parity (PPP) exchange rate adjusted prices are also presented. These adjusted prices were calculated by dividing national currency prices by the country-specific PPP exchange rate from year 2022 (35). The PPP relates to the price of a fixed set of consumables and goods across different countries, thus controlling for cost of living beyond the currency exchange rate.

To illustrate changes in price over time, three methods were employed:

1. For GDY, percentage changes in median prices over time were analysed in relation to the median price at initial extraction (as well as from the second extraction in Supplementary Table 1). For stifle surgeries, pyometra and caesarean sections prices were only analysed relative to the second extraction (the reason was that VP showed incomplete data at the first extraction).
2. In order to illustrate whether many clinics changed prices slightly, or a few clinics made substantial alterations of prices, or added prices were from new clinics; clinic prices for some diagnoses / procedures were plotted over time.
3. A sensitivity analysis for VP prices was conducted to examine how similar median prices and price increases were, comparing only new or updated prices to all prices that were found. This was done for caesarean sections and pyometra in cats and dogs, being receipt-based emergency procedures and hence only updated when owners provide receipts. The rationale was that if ‘all prices’ corresponded with ‘updated prices’ this would suggest that also receipt-based prices at VP could be updated frequently enough to provide a valid overall picture at a point in time. Thus, in the sensitivity analysis prices were not counted if an identical price from a specific clinic was found in the previous extraction.

### Validation

Cross-validation of extracted information was undertaken during the processing of the data through repeated browsing comparing web and VP prices for the same procedures in the same clinics. Manual web searching was not done systematically but frequently used as a means to check specific data entries. This was the first step to identify faulty outliers (but not to correct them). In the case of cat and dog GDY, percentage differences in price information were analysed when web and VP prices for the same procedure and clinic were identified during an extraction (termed internal validation, calculated as web price minus VP price, divided by web price). Method 3 in the above section (the sensitivity analysis) in the above section was also part of the internal validation. Furthermore, external validation, focusing on overall prices and price increases, was conducted by comparing prices from the web with those from VP. If the two sources corresponded, the credibility of the price retrieval as representative of prices available on the web was deemed high. It was considered that at least 10 clinics (i.e., 10 observations during each extraction, this figure selected as a practical cutoff), preferably more, should contribute to prices when generalising. External validation of prices for dog pyometra in SE was also done using information from a sample of 155 anonymised receipts from 2023/2024 (Odd-Einar Bruem, Svedea, personal communication) from over 80 different clinics. Prices were standardised using the same principles as at VP and categorised after clinic and regular-hours or after-hours. Included in the standardised fee were veterinary fee, consultation fee, blood samples including analysis, radiology or ultrasonography, surgery, anaesthesia, consumables, stationary care as normally performed at the clinic, and regular-hours emergency fee. The prices during after-hours included further emergency fees and additions that are made during after-hours (36, 37).

## Results

### Clinics

**Figure 1:**
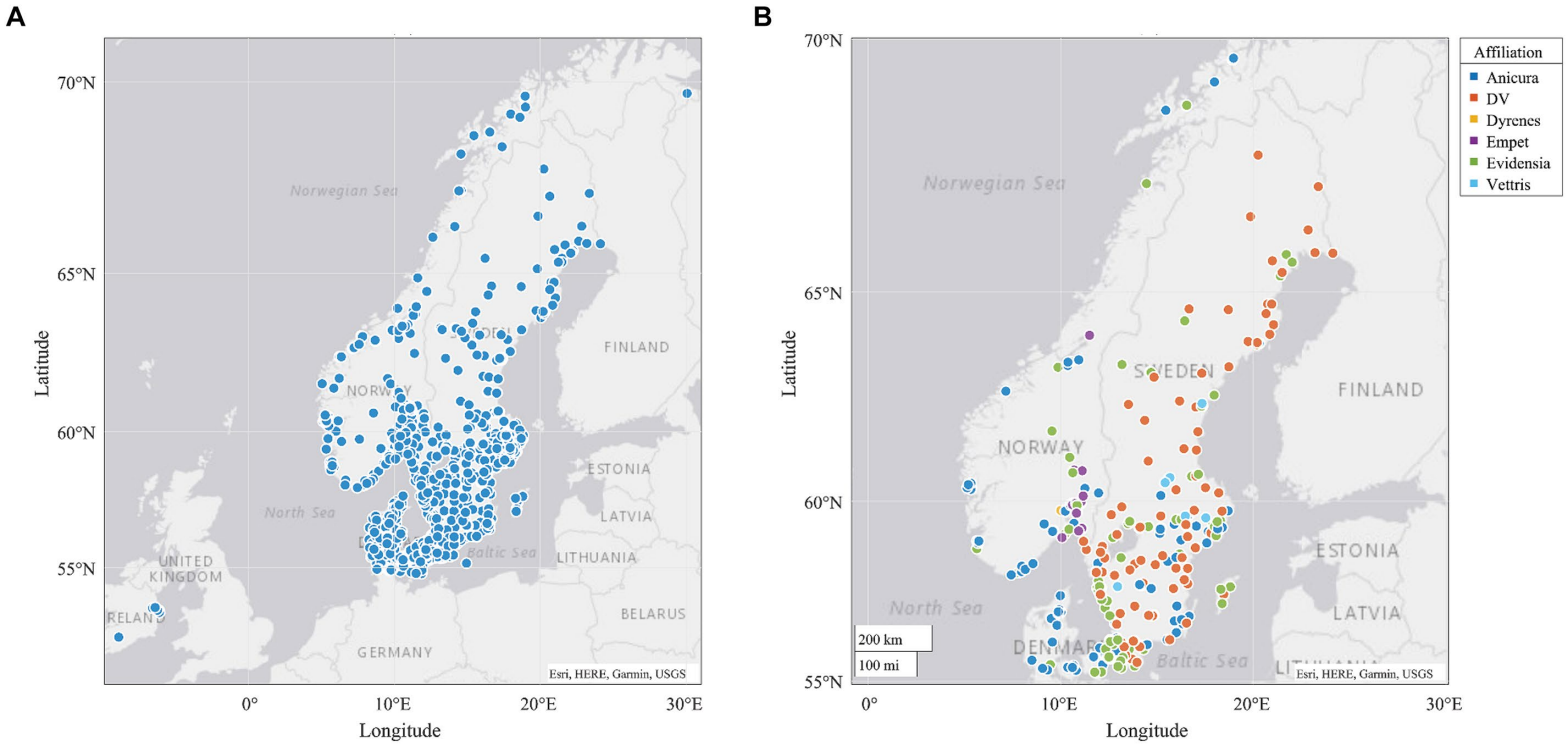
All clinics with price information and geo-coordinates **(A)** and the clinics with geo-coordinates found with common affiliations **(B)** from a study of prices for veterinary care from autumn 2022-winter 2023/2024.

Search results from the different sources yielded 9,625 unique clinics in SE, NO, DK, the UK and IR. Of these, 4,834 had at least one URL listed, and of these at least one URL could be retrieved for 4,466 clinics. These 4,466 clinics included 621 in SE, 428 in NO, 521 in DK, 2721 in UK, and 175 in IR. Information for the price field that was extracted from the web included data from 264 clinics in SE, 144 in NO, 224 in DK, 435 in the UK and 9 in IR, in total 1,076 clinics. However, the automated search and extraction methods applied did not yield useable price information from all these clinics; see further below for number of clinics with data for various procedures. VP had price information from 671 clinics in SE, 240 in NO and 193 in DK (all observations containing prices). On further analysis, some of the VP results also included non-veterinary clinics, e.g., physiotherapy ‘clinics’ (in SE 75 clinics, in DK 13 clinics, in NO zero clinics). Figure 1 displays the locations of the clinics with geo-coordinates, by affiliated company.

### Internal validation

During manual scrutinisation of data from VP, a number of prices (21 observations) were found to have been incorrectly registered at the VP site when compared to the prices on the website of a clinic or considered so when tabulated minimum and maximum values were compared to median values. For example, the price of hip radiology at a clinic during the first extraction was stated as 1 SEK when the real price at the clinic website was 1,050 SEK (these 21 prices were omitted when all the >150,000 price observations were tabulated).

Direct validation (Table 1) comparing web and VP (in this case prices that VP had collected from the web) prices for cat GDY in SE and NO was performed (i.e. for countries where there were ≥ 10 observations per extraction). In 13 of 24 cases the median differences were 0 %. The magnitude of the outliers were larger in NO than in SE. For dog GDY, direct validation was only possible for Swedish prices where there were ≥ 10 observations per extraction. In 6 of 12 cases the median difference was 0 % (Table 1). The impression when cross-checking results manually for web price vs. VP price (from the web) was that the automatic web price extraction performed in this project, compared to the prices at VP collected manually from the web, came with a rather large number of errors. There are many reasons for erroneous information in the data automatically extracted from the web. For example, postal codes, opening hours or telephone numbers were sometimes incorrectly identified as prices. However, these mistakes could easily be identified through manual scrutiny.

**Table 1:** Direct validation demonstrating differences (in percent) in price between the price at the veterinary clinic (web) and the price-comparison site (vetpris.se, VP) for cat and dog gonadectomy for two countries.

Species	Gender	Country	Time	N	Min	Median	Max
Cat	Male	NO	I	35	-50	0	46
			II	34	-47	4	35
			III	36	-43	1	56
			IV	41	-43	8	78
			V	46	-94	-1	53
			VI	44	-95	-1	17
		SE	I	42	-14	0	45
			II	42	-7	1	55
			III	37	0	0	55
			IV	37	0	0	55
			V	39	-4	0	55
			VI	40	-4	2	55
	Female	NO	I	33	-83	0	45
			II	32	-83	3	37
			III	35	-14	0	39
			IV	40	-83	2	55
			V	43	-83	5	45
			VI	42	-83	-1	9
		SE	I	38	-83	0	36
			II	37	-83	0	36
			III	33	-85	0	41
			IV	35	-85	0	48
			V	37	-85	0	46
			VI	39	-85	5	53
Dog	Male	SE	I	32	-14	0	23
			II	29	-13	0	25
			III	28	-25	0	31
			IV	31	-25	4	36
			V	33	-13	2	36
			VI	35	-13	3	43
	Female	SE	I	27	1	1	1
			II	26	-11	-1	-1
			III	26	-7	1	1
			IV	29	-11	0	0
			V	32	-12	0	0
			VI	32	-15	0	0

Extractions were done 6 times (Time), approximately every 3 months, during autumn 2022—winter 2023/2024. The figures are calculated as (web price minus VP-price) divided by VP-price (the VP price is originally web-based). Positive figures mean that web-prices were higher than VP-prices.

### External validation

There were 155 receipts provided for validation. For 48 included receipts it was clear that the visit belonged to a clinic found in the VP data, with a date that matched the extraction period and was complete enough to allow for standardisation. For regular-hours pyometra receipts (*n* = 24) the price listed at VP was below the standardised prices from the receipts in all but one case. For after-hours receipts (also *n* = 24) the VP price was lower than the validation price from the receipts in 17 instances. The median percentage difference [receipt price – VP price / receipt price] was 29% (range [−11%] – 56%) for regular-hours pyometra. For after-hours pyometra these figures were 9% (range [−44%] – 38%). The more extreme differences included a clinic that changed ownership (receipts included before and after, the VP price from ‘before’ was 54% of the validation price), one inadequate standardisation of a receipt received at VP, as well as a couple of VP prices that were outdated.

### Prices

**Figure 2:**
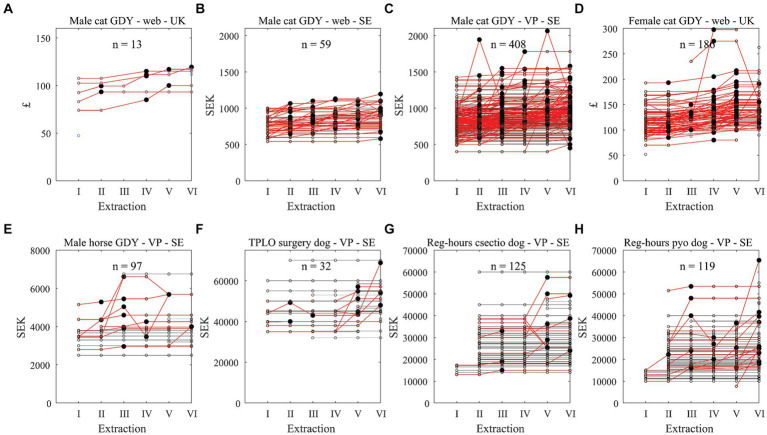
Prices through extraction time points by clinic, for various diagnoses and either web or vetpris (VP) and country **(A–H).** Black lines represent clinics that did not change the price. Red lines represent clinics that changed the price, with large black dots representing when changes took place. Prices are shown in original currency in order to enhance demonstration of whether stated prices changed through time. Data are from a study of prices for veterinary care from autumn 2022 - winter 2023/2024. The number of clinics with data within each plot are shown. GDY, gonadectomy; reg-hours, regular-hours; csectio, caesarean section; pyo, pyometra.

Magnitudes and changes of prices for GDY, stifle surgery, caesarean section and pyometra, are presented by source (clinic web & VP), country and time of extraction (Tables 2–6; Supplementary Table 1; Figure 2). In general, external validity increased as the number of clinics with price information increased. In Tables 1–6 only procedure/diagnoses with at least 10 clinics per extraction have been presented. Note however that the 10 clinics per extraction rule was ignored for ‘only new and updated’ prices. Supplementary Table 1 presents the same tabulations including procedure/diagnoses where there were at least 2 clinics contributing information. Tables 2–6 include median prices, but also minimum and maximum prices to address price ranges.

**Table 2:** Descriptive statistics of the prices (€) for gonadectomy of cats by country.

Source/country		Time									Change (%)	
			Male cats				Female cats				National currency	
			N	Min	**Median**	Max	N	Min	**Median**	Max	Male	Female
Web	NO	I	67	81	**123**	281	63	35	**235**	415		
		II	63	80	**124**	231	60	35	**242**	436	2	4
		III	50	94	**132**	225	48	176	**242**	325	16	11
		IV	56	72	**120**	196	54	31	**238**	393	10	14
		V	66	9	**148**	271	63	32	**269**	400	31	24
		VI	65	9	**146**	272	62	32	**258**	400	28	19
	SE	I	54	50	**74**	93	49	14	**131**	233		
		II	55	49	**71**	96	49	13	**135**	247	0	6
		III	47	48	**70**	97	44	13	**133**	244	0	6
		IV	48	46	**71**	96	46	13	**129**	190	5	8
		V	51	47	**74**	98	49	13	**136**	238	7	11
		VI	52	52	**79**	108	51	14	**152**	248	10	20
	UK						138	60	**136**	221		
							137	79	**134**	218	8	1
							19	100	**138**	267	9	2
							132	93	**151**	347	15	10
							150	89	**155**	331	30	17
							163	104	**160**	302	26	17
VP	DK	I	47	76	**136**	265	48	139	**226**	391		
		II	47	73	**131**	256	48	134	**219**	378	-2	-2
		III	61	72	**139**	253	62	132	**226**	349	4	1
		IV	93	69	**147**	253	94	132	**235**	352	9	6
		V	127	71	**152**	255	127	134	**238**	357	16	9
		VI	152	73	**152**	304	152	135	**240**	405	14	8
	NO	I	131	72	**121**	312	130	115	**230**	521		
		II	153	71	**124**	309	152	114	**228**	517	3	0
		III	206	67	**120**	323	205	106	**219**	484	7	2
		IV	206	64	**114**	313	205	101	**212**	478	6	3
		V	210	68	**132**	331	208	107	**241**	506	18	13
		VI	210	68	**134**	332	209	107	**250**	507	20	17
	SE	I	358	37	**70**	133	345	56	**126**	233		
		II	367	36	**69**	175	352	54	**127**	225	3	4
		III	376	35	**71**	137	361	53	**131**	244	7	9
		IV	370	34	**68**	151	354	51	**125**	233	7	9
		V	375	35	**72**	191	353	69	**130**	238	10	11
		VI	381	41	**77**	198	359	59	**137**	248	13	12

Values are missing for male dogs in the UK because ≥10 prices could be found during each extraction (Supplementary Table 1, see sheet ‘full versions of Tables 2–6’). Change in median price presented in national currencies, related to extraction I. Prices extracted from the web of veterinary clinics (web) and the price comparison site (vetpris.se, VP, the VP price is originally webbased) at 6 extractions (Time), approximately every 3 months during autumn 2022–winter 2023/2024. The standardised price at VP concerns a dog has its testes, or the uterus and the ovaries, or only the ovaries removed. The price includes that the procedure is performed during regular-hours including: veterinary fee, consultation fee, surgery, anaesthesia and consumables. Prices are averaged over weight ranged in case such were found.

**Table 3:** Descriptive statistics of the prices (€) for gonadectomy of dogs by country.

Source/ country		Time									Change (%)	
			Male dogs				Female dogs				National currency	
			N	Min	**Median**	Max	N	Min	**Median**	Max	Male	Female
Web	SE	I	34	265	**384**	754	30	535	**656**	884		
		II	34	256	**388**	728	31	517	**665**	967	5	5
		III	34	261	**386**	718	31	468	**653**	842	6	5
		IV	37	267	**377**	686	34	508	**636**	805	8	7
		V	39	273	**390**	702	37	474	**656**	824	9	7
		VI	41	284	**420**	730	37	493	**704**	894	13	11
	UK	I					85	250	**391**	744		
		II					83	231	**383**	730		0
		III										
		IV					72	333	**445**	699		12
		V					79	328	**461**	701		22
		VI					90	339	**463**	725		18
VP	DK	I	54	279	**385**	658	54	532	**706**	1117		
		II	54	270	**371**	635	54	514	**682**	1079	-2	-2
		III	66	199	**391**	698	66	412	**675**	1063	3	-3
		IV	92	200	**392**	700	90	413	**674**	1118	4	-3
		V	123	201	**396**	1828	121	416	**670**	1180	7	-1
		VI	147	203	**398**	707	145	418	**682**	1185	5	-2
	NO	I	29	207	**422**	773	23	544	**719**	1415		
		II	31	214	**428**	795	24	539	**802**	1457	2	12
		III	39	210	**437**	795	31	505	**802**	1457	11	20
		IV	36	200	**413**	755	28	480	**760**	1385	10	19
		V	36	211	**438**	796	28	506	**803**	1458	12	20
		VI	38	212	**440**	797	29	403	**805**	1462	12	21
	SE	I	321	186	**382**	611	290	419	**665**	1018		
		II	329	180	**391**	625	296	405	**676**	983	6	5
		III	338	171	**405**	617	300	416	**682**	968	11	8
		IV	334	164	**387**	606	295	426	**659**	926	11	9
		V	329	168	**396**	640	276	425	**668**	948	11	8
		VI	335	180	**412**	739	279	441	**707**	1000	11	10

Change in median price presented in national currencies, related to extraction I. Prices extracted from the price comparison site (vetpris.se, VP, the VP price is originally web-based) at 6 extractions (Time), approximately every 3 months during autumn 2022–winter 2023/2024. The standardised price at VP concerns a horse with both testes descended. The procedure takes place at home or at a clinic. Included are 30 km journey to the farm or a consultation fee, veterinary fee, sedation, local analgaesia and castration.

**Table 4:** Descriptive statistics of the prices (€) for gonadectomy of horses (during sedation and analgaesia) in Sweden.

					Change (%)
Time	N	Min	Median	Max	National currency
I	84	233	323	878	
II	86	225	391	882	25
III	87	222	586	895	90
IV	87	212	560	855	90
V	84	217	492	876	64
VI	85	225	512	910	64

Change in median price presented in national currency, related to extraction I. Prices extracted from the price-comparison site (vetpris.se, VP, the VP price is originally web-based) at 6 extractions (Time), approximately every 3 months, during autumn 2022 - winter 2023/2024. The standardised price at VP concerns a horse with both testes descended. The procedure takes place at home or at a clinic. Included are 30 km journey to the farm or a consultation fee, veterinary fee, sedation, local analgaesia and castration.

**Table 5:** Descriptive statistics of the prices (€) for stifle surgery procedures in dogs in Sweden.

						Change (%)
Procedure	Time	N	Min	Median	Max	National currency
TPLO	I	20	3,259	4,190	5,586	
	II	23	3,147	4,072	6,293	
	III	26	2,835	3,987	6,202	-1
	IV	27	2,710	3,812	5,929	-1
	V	30	2,774	4,225	6,069	8
	VI	30	2,883	4,505	6,307	10
TTA	I	11	2,607	3,259	4,186	
	II	13	2,517	3,147	4,042	
	III	16	2,484	2,990	4,430	-4
	IV	13	2,375	2,965	4,235	0
	V	15	2,431	3,035	4,335	0
	VI	15	2,526	3,154	4,505	0

Change in median price presented in national currency, here related to extraction II. Prices have been extracted from the price-comparison site (vetpris.se, VP, the VP price is mainly based on mail communication with clinics in this case) at 6 extractions (Time), approximately every 3 months, during autumn 2022 - winter 2023/2024. The standardised price at VP concerns a 30 kg dog with a confirmed diagnosis. Included are veterinary fee, surgery and stationary care. Tibial plateau levelling osteotomy (TPLO), tibial tuberosity advancement (TTA).

**Table 6:** Descriptive statistics of the prices (€) for caesarean section and pyometra in dogs, within regular-hours and after-hours in Sweden both for all prices, and for only new or updated prices.

										Change (%)	
		All prices				New/updated prices				National currency	
Diagnosis / procedure	Time	N	Min	**Median**	Max	N	Min	**Median**	Max	All prices	Upd. prices
Ceasarean section	I	10	1,210	**1,397**	1,616	10	1,210	**1,397**	1,616		
	II	86	1,169	**2,248**	5,394	76	1,598	**2,268**	5,394		
	III	91	1,240	**2,215**	5,316	8	1,329	**2060**	3,082	0	-8
	IV	86	1,186	**2,118**	5,082	1	2,965	**2,965**	2,965	0	39
	V	114	1,214	**2,196**	5,202	36	2,196	**2,196**	4,989	1	0
	VI	116	1,261	**2,282**	5,406	7	2,162	**3,222**	4,437	1	42
Ceasarean section	I	13	2,514	**3,575**	4,655	13	2,514	**3,575**	4,655		
After-hours	II	15	2,427	**3,956**	4,495	2	4,135	**4,180**	4,225		
	III	18	1949	**3,650**	4,873	3	1949	**3,101**	4,873	-6	-25
	IV	18	1863	**3,490**	4,235	1	3,889	**3,889**	3,889	-6	-1
	V	28	2,341	**3,842**	6,069	15	2,861	**3,842**	6,069	1	-5
	VI	31	2,433	**3,993**	7,767	4	3,644	**4,195**	7,767	1	0
Pyometra - uterine	I	13	931	**1,210**	1,404	13	931	**1,210**	1,404		
Inflammation	II	76	899	**1798**	4,628	65	1,384	**1978**	4,628		
Regular-hours	III	81	886	**1772**	4,733	10	1,418	**2,392**	4,733	0	23
	IV	78	847	**1779**	4,524	4	1,694	**2,147**	2,541	5	15
	V	109	666	**2,168**	4,631	34	666	**2,196**	3,176	25	15
	VI	115	901	**2,282**	5,892	16	1,622	**2,833**	5,892	27	43
Pyometra - uterine	I	14	2,328	**3,587**	5,027	14	2,328	**3,587**	5,027		
Inflammation	II	15	2,248	**3,731**	4,855	3	3,416	**4,315**	4,564		
After-hours	III	18	2,658	**4,016**	4,873	5	2,835	**4,430**	4,873	18	4
	IV	19	2,541	**3,896**	4,659	4	3,812	**4,254**	4,521	19	5
	V	29	2,601	**3,842**	6,176	15	3,768	**3,842**	6,176	15	-8
	VI	33	2,703	**3,993**	6,447	6	3,534	**4,920**	6,447	15	14

Change in median price presented in national currenyc, here related to extraction II. Data from extraction 1 has been included for completeness- but shall not be used for comparative purposes. Prices extracted/collected from the price-comparison site (vetpris.se, VP, the VP price is based on owner-submitted receipts) at 6 extractions (Time), approximately every 3 months, during autumn 2022 - winter 2023/2024. For both caesarean section and pyometra it is stipulated that the clinical course is uncomplicated in a dog of 30 kg. In the case of caesarean section there are 5 puppies. Included are veterinary fee, consultation fee, blood samples including analysis (only for pyometra), radiology (or ultrasonography), surgery, anaesthesia, consumables, stationary care as normally performed at the clinic (only for pyometra) and regular-hours emergency fee. The prices during after-hours include further emergency fees and additions that are made during after-hours.

For cat GDY (Table 2; Supplementary Table 1) the median web and VP prices (VP prices originally from the web) were generally similar. For male cat GDY, SE had lowest prices after 1 year (in fall year 2023 median for web and VP, respectively, was €74/€72), followed by the UK (few prices found, Supplementary Table 1), NO (€148/€132) and DK (VP €152). For female cat GDY, in SE (for web and VP, respectively, the prices were €136/€130) and UK (€155) had lower prices than NO (€269/€241) and DK (VP €238). For male cat GDY (Table 2) and male dog GDY (Table 3), in the UK few prices were found (because it was not possible to readily identify them in the collected data, see Supplementary Table 1 for actual numbers). The smallest median price in the fall of 2023 for male dogs was found in SE (for web and VP, respectively €390/€396) and DK (VP €396), whereas the price in NO was larger (VP €438). For female dog GDY, after 1 year the median web price in the UK was €461, followed by SE (for web and VP, respectively €656/€668) and DK (VP €670). NO (VP €803) had the largest mean price. Yearly changes in price [from first to fifth extraction] varied from −1% (female dogs in DK) to 31% increase (male cats in NO). Supplementary Figure 1 shows PPP-adjusted prices along with prices in Euro for the data in Tables 2, 3. Both measures suggest the same between-country differences. Note that while currency rates are continuous, PPP is set per year, which makes it difficult to compare price changes within a year between Euro and PPP-values.

For horse GDY (Table 4; Supplementary Table 1), there was only sufficient data from SE and only from VP (web price from VP), and 69.5% of the price entries found were from the country’s District Veterinary Officers (nationwide government-run ambulatory practice veterinarians, catering to farm animals, horses, and with smaller pet clinics, along with some horse clinics managed by the state). The median price fluctuated since first extraction but had increased with 64% in SEK by the fall of 2023. In this case the median prices were highest during the third and fourth extraction.

Prices for stifle surgery in dogs are presented separated by two (Table 5) or three (Supplementary Table 1) different surgical procedures. Only for VP in SE the number of observations was ≥10 during all extractions. Note that during the first extraction there were exactly 20 observations for TPLO, which suggests that the prices shown are the lowest of the ones reported to VP. Data from extraction I have been included for completeness- but shall not be used for comparative purposes. Price changes have been illustrated using the median price at the second extraction as the denominator. At the last extraction, median prices for TPLO were at €4,500 and for TTA close to €3,154. The largest price change at the end of the year was a 10% increase for TPLO. Information on stifle surgery prices had to a large degree been asked for by VP directly from the clinics (Olivia Schell at VP, personal information), as stifle surgery prices are seldom found on clinic websites.

VP prices for caesarean section and pyometra (receipt-based at VP) are presented in Table 6 and Supplementary Table 1, counting both all prices found at each extraction and prices with only new and updated prices for the sensitivity analysis. In SE and for dogs, most counts were large enough (≥10 observations) for interpretation, at least when all prices found were considered. Data from extraction I are again included, but the denominators for change calculations are from the second extraction. For regular-hours (day-time ‘emergency’ case) procedures median prices were ~ €2,300 for both caesarean section and pyometra in dogs at the last extraction from analysis of all prices. For the [new or updated prices the median prices were higher, ~€3,200 (caesarean section) and ~ €2,800 (pyometra)]. There is a large variation, the lowest price for regular-hours pyometra was €666 and the highest €5,892. For regular-hours caesarean section the range was €1,169 to €5,406. Using all prices, the results suggest that prices for regular-hours pyometra in dogs in SE increased with 27%. Using only new and updated prices, there was just over 40% increase in price for regular-hours pyometra and caesarean section. Pyometra performed after-hours showed a 15% increase in price. At the last extraction, the median prices for caesarean section and pyometra in dogs in SE were 57–59% higher when surgery was performed after-hours compared to during regular-hours (numerators and denominators from Table 6). The number of data points for NO and DK was low for these two procedures and was thus not included in the main analysis (but found in Supplementary Table 1).

In order to illustrate whether many clinics changed prices slightly, a few clinics made substantial alterations of prices, or added prices were from new clinics the price for some diagnoses/procedures were plotted over time for each clinic. Figures 2G,H show that increased VP-prices for regular-hours pyometra and caesarean section on dogs in SE, mainly depend on an added effect of more prices starting from the second extraction. This depends on the limited number of prices shown during the time of the first extraction. For regular-hours pyometra in SE, only 21 out of 119 clinics had their VP-price changed during the study time (specifically increases in prices except in 1 case a decrease and in another first a raise and then a decrease). For regular-hours caesarean section in SE, 12 of 125 clinics changed their standardised prices during the year (specifically increases in price except in two cases where the prices decreased). For male cat and horse GDY in SE VP-prices were updated relatively more often. For the 97 Swedish clinics with prices for horse GDY, 78 had updated prices updated at least once. Web prices for female cat GDY in the UK showed a steady increase in price over most of the clinics found (Figure 2D).

## Discussion

This study aimed to outline the prices of veterinary care for selected diagnoses/procedures for pets and horses across a handful of European countries over a one-year period. Additionally, it sought to explore the utility of web searching techniques in obtaining data and to verify sources online and through VP. Generally, and based on validated data, there was observed variation in prices across countries, with some notable increases over the year. For pet GDY, the results showed median price changes ranging from a decrease of 1% (female dogs in DK) to an increase of 31% (male cats in NO) over the year. The annual price escalations for regular-hours pyometra in SE were estimated at 27% and higher if only updated or new receipts were included. Price information for IR was only available from a limited number of clinics for each diagnosis/procedure (see examples in Supplementary Table 1). Regarding dog and cat GDY, the median web prices and the median VP prices were similar. Despite encountering several challenges, there is potential for future use of web searching and the VP site for discovering prices and calculating price changes. However, given the necessity for extensive data cleaning and validation, particularly of price information sourced from the web, and the dependency of receipt-based VP prices on timely submission by animal owners, enhanced price transparency from the veterinary clinics is imperative.

### Validation and comparison of web and VP prices

Both web and VP data (sourced from clinic websites) exhibited errors, often linked to significant outliers in prices (refer to Table 1; Supplementary Table 1). Manual cross-checking between clinic web homepages and VP data was crucial for data management, with a deliberate effort to avoid excessive over-correction. Errors were more prevalent in web data compared to VP data. The web-based approach to data collection failed to capture all relevant information, even when such information was found in the capture (e.g., male cat and male dog GDY in the UK), and included some erroneous entries, such as telephone numbers or hours mistakenly included in the price field. These errors stemmed from the diverse structures of web pages, necessitating adaptability in the search tool to accommodate various formats of price lists. Real-time validation during extraction was beyond the scope of this study.

Mistakes identified in VP data were generally limited to typos or in two cases to erroneous transfer of information from the actual receipt to the standardised format at VP (one example found through the external validation). Unlike a more automated search, VP allowed for the inclusion of additional web prices manually found, even those embedded within longer sentences on a web page. The VP approach also facilitated the staff’s understanding of each price’s context, indicating whether it represented the full price or required additional standard consultation fees. Veterinary clinics could independently manage their prices on VP, and prices labelled as ‘clinic prices’ underwent scrutiny by those clinics, particularly towards the latter part of the study period as VP gradually gained wider recognition among veterinarians and animal owners after its official launch in December 2022. It is highly likely that discrepancies between VP data and actual prices paid prompted some animal owners to contact VP, enhancing the likelihood of VP reporting accurate and reliable prices.

Observations revealed some discrepancies when utilising all available VP prices (for receipt-based procedures) and those with new or updated prices, as shown in Table 6. This was anticipated for two reasons. Firstly, in the light of the increasing public awareness, there may be a tendency for owners to increasingly submit receipts with relatively higher prices, elevating the median of the only new and updated prices presented. On the other hand some un-updated prices may be ‘outdated’ and therefore too low. The latter was found in the validation using insurance receipts for dog pyometra—when one privately-owned clinic with rather low prices changed ownership to a private equity firm, prices for regular-hours pyometra doubled. The observed increases aligned well for regular-hours pyometra, after-hours pyometra, and out-of-hours caesarean section, but not for regular-hours caesarean section. The results suggest that the general price trend seen in VP data for receipt-based procedures could be reasonable, but likely somewhat conservative, when using a short time window, as in the current study. Recent data from Denmark indicate that prices were updated twice a year (38).

The VP website serves as a valuable tool for owners seeking approximate price estimates for various procedures in SE, NO and DK. For elective and well-defined procedures, owners can generally rely on the clinic’s price listed on the VP site, which typically matches the amount they can expect to pay. Once a clinic is selected, owners can often easily verify this information by visiting the clinic’s website. However, for more urgent issues, prices are seldom available on clinic websites [except for the country’s District Veterinary Officers (39), see below]. We speculate that the standardised price at a targeted clinic found on VP would in general be somewhat lower (from external validation using insurance receipts, the median VP-price was 29% lower for regular-hours pyometra) than what an owner would pay if they took their animal to that clinic, assuming no medical complications arise. Nevertheless, for prices not found on clinic websites, VP staff rely on a continuous influx of receipts from owners, highlighting the importance of owner involvement (i.e., citizen participation) in gathering information. The frequency of price updates may vary based on the nature of the procedure. Although billing date information is not reported at VP due to confidentiality concerns, calendar year has been included since December 28, 2023, to provide website visitors with a time reference for new entries. In general, it is rare to find prices on the web for emergency procedures (one exception in SE being the country’s District Veterinary Officers) (39), making price comparisons between providers difficult. Veterinary clinics determine if they are willing to provide detailed estimates of total costs before admission or after diagnostic workup for emergency situations, further complicating comparisons among caregivers. Veterinary clinics are often reluctant to suggest what prices could amount to before having examined the patient. We believe the main reason is that the price to a great deal will depend on the exact diagnosis, comorbidities and the general condition of the animal. However, some clinics use more-fixed fees for concrete procedures and others put every utensil on the receipt (as observed in the external validation of the 155 pyometra receipts).

### Price comparisons

We attempted to focus on several procedures with the aim of obtaining valid, comparable results across countries and with relevance to similar procedures within each country. Magnitudes of prices were prioritised over price changes as the time window was short, only 12–15 months. We note that even though prices for male cat and male dog GDY from the UK were found in the dataset (found during “manual” scrutiny of data), few were captured for analysis (Tables 2, 3; Supplementary Table 1). It was deemed impossible to securely filter those out (correct species and correct sex) from the dataset, even when using extensive regular expressions (Supplementary Data Sheet 1; sheet SAScode castration). The data for GDY were represented by a large number of clinics in SE, with most other countries having at least 10 observations per extraction, which was deemed the minimum for interpretation. Considerable variation in prices was observed at each point in time across all countries, with clear differences in median prices between countries. In the autumn of 2023 (1 year after the initial extraction), SE had the most affordable male cat GDY (for web and VP, respectively €74/ €72), while SE and UK were collectively the cheapest for female cat GDY (SE web/VP €136/130, UK web €155). The highest prices for cat GDY were observed in DK (males €152, females €238) and NO (males approx. €140, females ≥ €241). The price for female dog GDY ranged from €461 (UK) to €803 (NO) at the penultimate extraction. Prices of GDY may have been subsidised relative to other prices, as previously found (40), which could vary across countries. For GDY there was no direct relation between price and prevalence of insurance. This may be due to that GDY is not generally covered by insurance and thus prices are lower in Sweden (GDY may be covered by insurance if part of essential treatment of disease). The reason for low prices on GDY in the UK may be that GDY prices has been kept low for the greater good, to keep the overpopulation low, for quite some time. However, to compare prices between countries more generally necessitates price information on a large number of procedures. Considerable variation in standardised prices was also observed within each country. However, the variation would likely have been even larger if actual amounts on receipts had been directly used. In SE, prices for emergency procedures for dogs differed substantially between clinics, with the highest price being up to five times higher than the lowest. Median standardised prices for regular-hours caesarean section/pyometra in SE were approximately €2,300, rising to nearly €4,000 for after-hours procedures in the final extraction.

Published data on prices and price changes over time for veterinary care procedures in small animals or horses are scarce. However, one recent study from France (41) explored the turnover and time allocated to procedures at veterinary clinics, revealing positive associations between the characteristics of a practice, turnover, and prices of different procedures. An older study from SE showed a 41% increase in the cost of insuring horses for veterinary care between 1997 and 2004, outpacing the 10% increase in consumer price index (CPI) during the same period (23). While these insured costs parallel healthcare prices for health issues, they exclude prophylactic costs such as GDY. Furthermore, in SE, 8 out of 10 veterinarian visits were booked in advance in 2022 (42), indicating a slight decrease from previous years and implying that 2 out of 10 visits represent acute problems. However, maintaining clinic workforce and veterinarians’ salaries, which remain relatively low, pose challenges in SE (43) and other parts of the world, suggesting rising staff salary costs are unlikely to be the main driver of increasing prices.

### Owners, veterinarians, and price discussions

A focus group study conducted in Canada in 2004 explored the perceptions of animal owners and veterinarians regarding their communication on prices (40). Owners expressed a desire for clear explanations of prices and alternatives, while veterinarians believed that owners primarily wanted to be directed on what to do. Interestingly, recent findings in SE indicate that it is uncommon for owners to inquire about prices before initiating treatment, possibly due to the high prevalence of animal insurance (44).

Amidst poor price transparency from veterinary care providers worldwide, one solution may lie in the increased awareness and use of platforms like VP for accessing timely price information. In the UK, price comparisons starting with consultation fees, were recently added to an existing site listing veterinary clinics (45). The VP price comparison site aligns with a recent Swedish Government Official Report advocating for transparency in the veterinary care sector (1). As a result, the organisation of the District Veterinary Officers in SE has made their complete price list available online in 2023 (39). The challenge prompts a call on the veterinary profession to address price transparency with clarity. Failure to do so may lead to increased governmental initiatives, as seen in the UK (2), aimed at examining the economic viability of the veterinary sector to ensure accessible and affordable care for animals. Another way would be for veterinary clinics to publish prices on the average prices, or an interval of prices, clients have previously paid for more common procedures, e.g., in the last year. In this way the displayed prices would be directly related to the final prices on past receipts (a degree of manual audit could be necessary for the clinics). It is however possible that clinics with more transparent and higher prices could make owners choose other clinics, even those with less transparent prices. Thus it would be difficult for individual clinics to do this unless a majority of clinics did the same. Further, a comprehensive understanding of the economic landscape of the veterinary care sector requires data from various sources, including veterinary clinics, insurance data, owner receipts, company turnovers, and surveys organised by country administration. However, all of these sources are not publicly available and it would require cooperation between scientists and stakeholders. Conducting careful research comparing results from different sources, each one with different benefits and limitations, will enhance the understanding and analysis of the veterinary market, guiding decisions beneficial to animals and society.

The countries included in this study were selected based on their similar standard of living, despite underlying economic differences (e.g., the experiences in the UK following Brexit) (46). The COVID-19 pandemic has also impacted countries, with many people acquiring pets during lockdown periods (47). However, discussing the interrelation of these factors is beyond the scope of this study. During the study period, the CPI in the respective countries varied. Specifically, it was 9.7% in SE (May 2023) (48), 6.7% in NO (May 2023) (49), 2.9% in DK (May 2023) (50), 7.9% in the UK (May 2023) (51) and 6.6% in IR (May 2023) (52). Interpretation from the PPP-adjusted measures parallel those using Euro for between-country comparisons.

Statistics Sweden recently reported a 25% increase in veterinary care prices during the year (53). However, the transparency of the extraction procedures for these statistics was questioned (54), urging caution in generalising the results. In the Nordic countries, the increase in prices of elective procedures exceeded the CPI and in SE, also the price for pyometra surgery was higher than CPI (at a 27% median increase compared to 5.4% CPI in SE; Jan 2024) (48).

This study does not address the reasons behind changes in veterinary prices or whether prices are generally higher or lower in some countries compared to others. Furthermore, it does not explore changes in the quality of veterinary services or whether higher prices are related to the size of the clinics as larger clinics will generally have more advanced equipment than smaller clinics. The discourse on optimal veterinary care is often influenced by the perception that pet owners today seek the best possible care for their animals. There is little actual evidence on outcomes from different levels of care. The ongoing debate emphasizes the idea that relying on the most expensive procedures to save individuals will not always be ‘the best’ and may ultimately lead to a decline in overall population well-being (13, 25). This has been formally addressed under umbrella concepts such as spectrum of care in North-America (13) and contextualised care in the UK (25), both concepts suggesting fine-tuned shared-decision making is needed, emphasising that owners should engage in choosing level of care/service. The veterinary profession must increasingly consider that care can be optimally targeted for the individual and be sustainable, making it possible for more animals to get adequate care, thus taking account of patient determinants and owner resources. Unpublished insurance data (the same data as used in the external validation for pyometra prices, *n* = 155) suggests that in 92% of the cases imaging of the uterus was performed for diagnosis, and in 70% of the cases some type of blood sampling was performed before treatment (personal communication Odd-Einar Bruem). The degree to which these diagnostic procedures actually help improve the outcome for the patient population has not been investigated.

### Limitations

This study has several practical limitations, many of which have been previously addressed. The web searching tool we utilised requires updating for future use, and specific measures can be implemented to improve the ease and transparency of data extraction from the resulting file. Many of the challenges related to the lack of similarity of the clinic websites. The procedures and diagnoses included in this study were selected to reflect various aspects of veterinary care, for which a sufficient number of prices could be extracted at each point in time. Although there was an attempt to filter out standard and emergency consultation fees, the results were challenging to interpret, and thus, they have not been presented here (for prices on such fees, we refer to Supplementary Data Sheet 1 and the VP-tags from vetpris.se, which indicate the contents of each price). While we aimed to thoroughly examine equine-related prices, particularly on the web, this proved difficult due to an insufficient number of observations. There is a risk that median receipt-based prices at VP are biased upwards if animal owners tend to submit receipts from clinics with relatively higher charges. To mitigate this effect, we have presented minimum, median, and maximum values to represent prices. Additional statistics, including means, are provided in Supplementary Data Sheet 1. In the authors’ opinion, it is unlikely that the changes observed during the study period are biased, as the inclination to submit receipts from various sources is unlikely to have changed over the year. Estimates of price changes were not associated with measures of spread; hence, no confidence intervals were produced. However, from the tables, it can be observed that changes were seldom linear over time, making most measures of change sensitive to variation, given the low number of observations from a market with significant inherent price variation across clinics and over time. One notable aspect of the study is that it only covers a short period, and to analyse changes over time rather than just magnitudes, data spanning longer time periods are required.

## Conclusion

This study describes prices of veterinary care for selected diagnoses/procedures for pets and horses in a few European countries that can be used as comparisons today and forward, and explores the use of web searching techniques to find and validate data. In lack of other available data sources we used web-searching. This research may motivate stakeholders interested in price transparency to provide data to improve price precision. The web searching tools required both automatic and manual validation, revealing numerous errors. While data were available for several countries, they were limited in others (such as the UK and IR), mainly due to fewer clinics providing price information. The VP site served as a valuable source of data for comparison and supplementation to the web search data, partly due to VP’s manual procedures. Collaboration among VP staff, animal owners, practitioners, and researchers in building a comprehensive database of prices could enhance this initiative’s value even further. Owners sharing receipt information may be the most viable way to ascertain actual prices. The price information gathered provides evidence of increasing prices and significant differences across countries. Further research on this topic is warranted. However, that would require increased transparency from the veterinary business community on prices as well as branding clinics that are part of larger corporate entities. The increased transparency might also help to alleviate confusion and frustration among owners seeking accessible and affordable care for their animals.

## Data availability statement

The datasets presented in this article are not readily available because the raw data may contain secondary personal identifiers and thus are not allowed according to Swedish law and GDPR to be public. As stated in the manuscript only two authors are allowed to handle those data. De-identified data could be shared with a third party upon reasonable request. Extensive supplements have been included to share as much data as possible. Requests to access the datasets should be directed to agneta.egenvall@slu.se.

## Author contributions

AE: Conceptualization, Data curation, Formal analysis, Funding acquisition, Investigation, Methodology, Project administration, Software, Validation, Visualization, Writing – original draft, Writing – review & editing. OH: Writing – original draft, Writing – review & editing, Conceptualization, Investigation, Methodology. RH: Writing – original draft, Writing – review & editing, Formal analysis. PV: Methodology, Writing – original draft, Writing – review & editing, Conceptualization. PA: Writing – original draft, Writing – review & editing, Methodology, Investigation. CL: Conceptualization, Writing – original draft, Writing – review & editing. AB: Conceptualization, Writing – original draft, Writing – review & editing, Data curation, Investigation, Methodology, Software. BB: Conceptualization, Writing – original draft, Writing – review & editing.
