# Correction: Prices for veterinary care of dogs, cats and horses in selected countries in Europe

**DOI:** 10.3389/fvets.2025.1694780

**Published:** 2025-10-17

**Authors:** Agneta Egenvall, Odd V. Höglund, Ruben Hoffman, Paul S. Valle, Pia Haubro Andersen, Cecilia Lönnell, Anna Byström, Brenda N. Bonnett

**Affiliations:** ^1^Agneta Egenvall, Odd Höglund, Department of Clinical Sciences, Faculty of Veterinary Medicine and Animal Husbandry, Swedish University of Agricultural Sciences, Uppsala, Sweden; ^2^Ruben Hoffman, Department of Economics, Faculty of Natural Resources and Agricultural Sciences, Swedish University of Agricultural Sciences, Uppsala, Sweden; ^3^Paul S. Valle, Tornes i R, Norway; ^4^Pia Haubro Andersen, Department of Anatomy, Physiology and Biochemistry (AFB); Division of Anatomy and Physiology, Swedish University of Agricultural Sciences, Uppsala, Sweden; ^5^Cecilia Lönnell, Tequi, Stockholm, Sweden; ^6^Anna Byström, Department of Applied Animal Science and Welfare, Swedish University of Agricultural Sciences, Uppsala, Sweden; ^7^Brenda N Bonnett, B Bonnett Consulting, Georgian Bluffs, ON, Canada

**Keywords:** animal insurance, corporate, inflation, veterinarian, veterinary care prices, animal health economics, price transparency, consumer

There was a mistake in Table 1 as published. There was a minor problem with the exchange rates for the Norwegian prices from the price comparison site. The conclusions and general reasoning remain identical. The corrected Table 1 appears below.

**Table 1:** Direct validation demonstrating differences (in percent) in price between the price at the veterinary clinic (web) and the price-comparison site (vetpris.se, VP) for cat and dog gonadectomy for two countries.

**Species**	**Gender**	**Country**	**Time**	***N***	**Min**	**Median**	**Max**
Cat	Male	NO	I	35	−50	0	46
			II	34	−47	4	35
			III	36	−43	1	56
			IV	41	−43	8	78
			V	46	−94	−1	53
			VI	44	−95	−1	17
		SE	I	42	−14	0	45
			II	42	−7	1	55
			III	37	0	0	55
			IV	37	0	0	55
			V	39	−4	0	55
			VI	40	−4	2	55
	Female	NO	I	33	−83	0	45
			II	32	−83	3	37
			III	35	−14	0	39
			IV	40	−83	2	55
			V	43	−83	5	45
			VI	42	−83	−1	9
		SE	I	38	−83	0	36
			II	37	−83	0	36
			III	33	−85	0	41
			IV	35	−85	0	48
			V	37	−85	0	46
			VI	39	−85	5	53
Dog	Male	SE	I	32	−14	0	23
			II	29	−13	0	25
			III	28	−25	0	31
			IV	31	−25	4	36
			V	33	−13	2	36
			VI	35	−13	3	43
	Female	SE	I	27	1	1	1
			II	26	−11	−1	−1
			III	26	−7	1	1
			IV	29	−11	0	0
			V	32	−12	0	0
			VI	32	−15	0	0

There was a mistake in Table 2 as published. There was a major problem with the exchange rate from the Danish prices from the price comparison site and minor problem with the exchange rates for the Norwegian prices from the price comparison site. The conclusions and general reasoning are moderately different for the Danish prices while they remain identical for the Norwegian prices. The corrected Table 2 appears below.

**Table 2:** Descriptive statistics of the prices (€) for gonadectomy of cats by country.

**Source/country**		**Time**	**Male cats**				**Female cats**				**Change (%)**	
											**National currency**	
			***N***	**Min**	**Median**	**Max**	***N***	**Min**	**Median**	**Max**	**Male**	**Female**
Web	NO	I	67	81	**123**	281	63	35	**235**	415		
		II	63	80	**124**	231	60	35	**242**	436	2	4
		III	50	94	**132**	225	48	176	**242**	325	16	11
		IV	56	72	**120**	196	54	31	**238**	393	10	14
		V	66	9	**148**	271	63	32	**269**	400	31	24
		VI	65	9	**146**	272	62	32	**258**	400	28	19
	SE	I	54	50	**74**	93	49	14	**131**	233		
		II	55	49	**71**	96	49	13	**135**	247	0	6
		III	47	48	**70**	97	44	13	**133**	244	0	6
		IV	48	46	**71**	96	46	13	**129**	190	5	8
		V	51	47	**74**	98	49	13	**136**	238	7	11
		VI	52	52	**79**	108	51	14	**152**	248	10	20
	UK						138	60	**136**	221		
							137	79	**134**	218		1
							19	100	**138**	267		2
							132	93	**151**	347		10
							150	89	**155**	331		17
							163	104	**160**	302		17
VP	DK	I	47	76	**136**	265	48	139	**226**	391		
		II	47	73	**131**	256	48	134	**219**	378	−2	−2
		III	61	72	**139**	253	62	132	**226**	349	4	1
		IV	94	69	**147**	253	94	132	**235**	352	9	6
		V	127	71	**152**	255	127	134	**238**	357	16	9
		VI	153	73	**152**	304	153	135	**240**	405	14	8
	NO	I	131	72	**121**	312	130	115	**230**	521		
		II	153	71	**124**	309	152	114	**228**	517	3	0
		III	206	67	**120**	323	205	106	**219**	484	7	2
		IV	206	64	**114**	313	205	101	**212**	478	6	3
		V	210	68	**132**	331	208	107	**241**	506	18	13
		VI	210	68	**134**	332	209	107	**250**	507	20	17
	SE	I	358	37	**70**	133	345	56	**126**	233		
		II	367	36	**69**	175	352	54	**127**	225	3	4
		III	376	35	**71**	137	361	53	**131**	244	7	9
		IV	370	34	**68**	151	354	51	**125**	233	7	9
		V	375	35	**72**	191	353	69	**130**	238	10	11
		VI	381	41	**77**	198	359	59	**137**	248	13	12

Values are missing for male dogs in the UK because ≥10 prices could be found during each extraction (Supplementary Table 1, see sheet ‘full versions of Tables 2–6'). Change in median price presented in national currencies, related to extraction I. Prices extracted from the web of veterinary clinics (web) and the price comparison site (vetpris.se, VP, the VP price is originally web-based) at 6 extractions (Time), approximately every 3 months during autumn 2022–winter 2023/2024. The standardised price at VP concerns a dog has its testes, or the uterus and the ovaries, or only the ovaries removed. The price includes that the procedure is performed during regular-hours including: veterinary fee, consultation fee, surgery, anaesthesia and consumables. Prices are averaged over weight ranged in case such were found.

There was a mistake in Table 3 as published. There was a major problem with the exchange rate from the Danish prices from the price comparison site and minor problem with the exchange rates for the Norwegian prices from the price comparison site. The conclusions and general reasoning are moderately different for the Danish prices while they remain identical for the Norwegian prices. The corrected Table 3 appears below.

**Table 3:** Descriptive statistics of the prices (€) for gonadectomy of dogs by country.

**Source/country**		**Time**	**Male dogs**				**Female dogs**				**Change (%)**	
											**National currency**	
			***N***	**Min**	**Median**	**Max**	***N***	**Min**	**Median**	**Max**	**Male**	**Female**
Web	SE	I	34	265	**384**	384	30	535	**656**	884		
		II	34	256	**388**	388	31	517	**665**	967	5	5
		III	34	261	**386**	386	31	468	**653**	842	6	5
		IV	37	267	**377**	377	34	508	**636**	805	8	7
		V	39	273	**390**	390	37	474	**656**	824	9	7
		VI	41	284	**420**	420	37	493	**704**	894	13	11
	UK	I					85	250	**391**	744		
		II					83	231	**383**	730		0
		III										
		IV					72	333	**445**	699		12
		V					79	328	**461**	701		22
		VI					90	339	**463**	725		18
VP	DK	I	54	279	**385**	658	54	532	**706**	1,117		
		II	54	270	**371**	635	54	514	**682**	1,079	−2	−2
		III	66	199	**391**	698	66	412	**675**	1,063	3	−3
		IV	92	200	**392**	700	90	413	**674**	1,118	4	−3
		V	123	201	**396**	1,828	121	416	**670**	1,180	7	−1
		VI	147	203	**398**	707	145	418	**682**	1,185	5	−2
	NO	I	29	207	**422**	773	23	544	**719**	1,415		
		II	31	214	**428**	795	24	539	**802**	1,457	2	12
		III	39	210	**437**	795	31	505	**802**	1,457	11	20
		IV	36	200	**413**	755	28	480	**760**	1,385	10	19
		V	36	211	**438**	796	28	506	**803**	1,458	12	20
		VI	38	212	**440**	797	29	403	**805**	1,462	12	21
	SE	I	321	186	**382**	382	290	419	**665**	1,018		
		II	329	180	**391**	391	296	405	**676**	983	6	5
		III	338	171	**405**	405	300	416	**682**	968	11	8
		IV	334	164	**387**	387	295	426	**659**	926	11	9
		V	329	168	**396**	396	276	425	**668**	948	11	8
		VI	335	180	**412**	412	279	441	**707**	1,000	11	10

Change in median price presented in national currencies, related to extraction I. Prices extracted from the price comparison site (vetpris.se, VP, the VP price is originally web-based) at 6 extractions (Time), approximately every 3 months during autumn 2022–winter 2023/2024. The standardised price at VP concerns a horse with both testes descended. The procedure takes place at home or at a clinic. Included are 30 km journey to the farm or a consultation fee, veterinary fee, sedation, local analgaesia and castration.

Supplemental material Supplemental Figure 1 was omitted. The file(s) have now been published.

File Presentation 1.pdf was erroneously published with the original version of this paper. The file has now been removed.

File Data Sheet 1.xlsx was erroneously published with the original version of this paper. The file has now been removed.

File Supplementary Table 1 was erroneously published with the original version of this paper. The file has been replaced with Supplementary Data Sheet 1.

File Supplementary Table 2 was erroneously published with the original version of this paper. The file has been replaced with Supplementary Data Sheet 2.

File Supplementary Table 3 was erroneously published with the original version of this paper. The file has now been replaced with Supplementary Table 1.

The Abstract included an exchange rate error within the **Results** section. This previously read: “By October 2023, median prices for male cat GDY ranged from €72 (SE) to €230 (DK), and €130 (SE) to €361 (DK) for females; for dog GDY from €390 (SE) to €599 (DK) for males, and €461 (UK) to €1015 (DK) for females. Across sources, median prices for cat and dog GDY increased by 2–24% over a year for procedures with at least 10 clinics per extraction.” This has been corrected to read:

By October 2023, median prices for male cat GDY ranged from €72 (SE) to €152 (DK), and €130 (SE) to €269 (NO) for females; for dog GDY from €390 (SE) to €438 (NO) for males, and €461 (UK) to €803 (NO) for females. Across sources, median prices for cat and dog GDY varied from a decrease of 1% to an increase of 31% over a year for procedures with at least 10 clinics per extraction.

A correction has been made to **Results**, *Prices* section, second paragraph. It previously stated: “For cat GDY (Table 2; Supplementary Table 1) the median web and VP prices (VP prices originally from the web) were generally similar. For male cat GDY, SE had lowest prices after 1 year (in fall year 2023 median for web and VP, respectively, was €74/€72), followed by the UK (few prices found, Supplementary Table 1), NO (€148/€135) and DK (VP €230). For female cat GDY, in SE (for web and VP, respectively, the prices were €136/€130) and UK (€155) had lower prices than NO (€269/€247) and DK (VP €361). For male cat GDY (Table 2) and male dog GDY (Table 3), in the UK few prices were found (because it was not possible to readily identify them in the collected data, see Supplementary Table 1 for actual numbers). The smallest median price in the fall of 2023 for male dogs was found in SE (for web and VP, respectively €390/€396), followed by NO (VP €449) and DK (VP €599). For female dog GDY, after 1 year the median web price in the UK was €461, followed by SE (for web and VP, respectively €656/€668), NO (VP €823) and DK (VP €1,015). Yearly changes in price [from first to fifth extraction] varied from 2% (female dogs in DK) to 24% increase (female cats in NO). Supplementary Figure 1 shows PPP-adjusted prices along with prices in Euro for the data in Tables 2, 3. Both measures suggest the same between-country differences. Note that while currency rates are continuous, PPP is set per year, which makes it difficult to compare price changes within a year between Euro and PPP-values.” This has been corrected to:

For cat GDY (Table 2; Supplementary Table 1) the median web and VP prices (VP prices originally from the web) were generally similar. For male cat GDY, SE had lowest prices after 1 year (in fall year 2023 median for web and VP, respectively, was €74/€72), followed by the UK (few prices found, Supplementary Table 1), NO (€148/€132) and DK (VP €152). For female cat GDY, in SE (for web and VP, respectively, the prices were €136/€130) and UK (€155) had lower prices than NO (€269/€241) and DK (VP €238). For male cat GDY (Table 2) and male dog GDY (Table 3), in the UK few prices were found (because it was not possible to readily identify them in the collected data, see Supplementary Table 1 for actual numbers). The smallest median price in the fall of 2023 for male dogs was found in SE (for web and VP, respectively €390/€396) and DK (VP €396), whereas the price in NO was larger (VP €438). For female dog GDY, after 1 year the median web price in the UK was €461, followed by SE (for web and VP, respectively €656/€668) and DK (VP €670). NO (VP €803) had the largest mean price. Yearly changes in price [from first to fifth extraction] varied from −1% (female dogs in DK) to 31% increase (male cats in NO). Supplementary Figure 1 shows PPP-adjusted prices along with prices in Euro for the data in Tables 2, 3. Both measures suggest the same between-country differences. Note that while currency rates are continuous, PPP is set per year, which makes it difficult to compare price changes within a year between Euro and PPP-values.

A correction has been made to **Discussion**, first paragraph: “For pet GDY, the results showed median price hikes ranging from 2% (female dogs in DK) to 24% (female cats in NO) over the year.” This has been corrected to:

For pet GDY, the results showed median price changes ranging from a decrease of 1% (female dogs in DK) to an increase of 31% (male cats in NO) over the year.

A correction has been made to the **Discussion**, *Price comparisons*, section, First paragraph. The incorrect paragraph reads as follows: “We attempted to focus on several procedures with the aim of obtaining valid, comparable results across countries and with relevance to similar procedures within each country. Magnitudes of prices were prioritised over price changes as the time window was short, only 12–15 months. We note that even though prices for male cat and male dog GDY from the UK were found in the dataset (found during ‘manual' scrutiny of data), few were captured for analysis (Tables 2, 3; Supplementary Table 1). It was deemed impossible to securely filter those out (correct species and correct sex) from the dataset, even when using extensive regular expressions (Supplementary Data 1; sheet SAScode castration). The data for GDY were represented by a large number of clinics in SE, with most other countries having at least 10 observations per extraction, which was deemed the minimum for interpretation. Considerable variation in prices was observed at each point in time across all countries, with clear differences in median prices between countries. In the autumn of 2023 (1 year after the initial extraction), SE had the most affordable male cat GDY (for web and VP, respectively €74/ €72), while SE and UK were collectively the cheapest for female cat GDY (SE web/VP €136/130, UK web €155). The highest prices for cat GDY were observed in NO (males approx. €140, females ≥ €247) and DK (males €230, females/€361). The price for female dog GDY ranged from €461 (UK) to €1015 (DK) at the penultimate extraction. Prices of GDY may have been subsidised relative to other prices, as previously found (40), which could vary across countries. For GDY there was no direct relation between price and prevalence of insurance. This may be due to that GDY is not generally covered by insurance and thus prices are lower in Sweden (GDY may be covered by insurance if part of essential treatment of disease). The reason for low prices on GDY in the UK may be that GDY prices has been kept low for the greater good, to keep the overpopulation low, for quite some time. However, to compare prices between countries more generally necessitates price information on a large number of procedures. Considerable variation in standardised prices was also observed within each country. However, the variation would likely have been even larger if actual amounts on receipts had been directly used. In SE, prices for emergency procedures for dogs differed substantially between clinics, with the highest price being up to 5 times higher than the lowest. Median standardised prices for regular-hours caesarean section/pyometra in SE were approximately €2,300, rising to nearly €4,000 for after-hours procedures in the final extraction.” This paragraph has been corrected to:

We attempted to focus on several procedures with the aim of obtaining valid, comparable results across countries and with relevance to similar procedures within each country. Magnitudes of prices were prioritised over price changes as the time window was short, only 12–15 months. We note that even though prices for male cat and male dog GDY from the UK were found in the dataset (found during “manual” scrutiny of data), few were captured for analysis (Tables 2, 3; Supplementary Table 1). It was deemed impossible to securely filter those out (correct species and correct sex) from the dataset, even when using extensive regular expressions (Supplementary Data 1; sheet SAScode castration). The data for GDY were represented by a large number of clinics in SE, with most other countries having at least 10 observations per extraction, which was deemed the minimum for interpretation. Considerable variation in prices was observed at each point in time across all countries, with clear differences in median prices between countries. In the autumn of 2023 (1 year after the initial extraction), SE had the most affordable male cat GDY (for web and VP, respectively €74/ €72), while SE and UK were collectively the cheapest for female cat GDY (SE web/VP €136/130, UK web €155). The highest prices for cat GDY were observed in DK (males €152, females €238) and NO (males approx. €140, females ≥€241). The price for female dog GDY ranged from €461 (UK) to €803 (NO) at the penultimate extraction. Prices of GDY may have been subsidised relative to other prices, as previously found (40), which could vary across countries. For GDY there was no direct relation between price and prevalence of insurance. This may be due to that GDY is not generally covered by insurance and thus prices are lower in Sweden (GDY may be covered by insurance if part of essential treatment of disease). The reason for low prices on GDY in the UK may be that GDY prices has been kept low for the greater good, to keep the overpopulation low, for quite some time. However, to compare prices between countries more generally necessitates price information on a large number of procedures. Considerable variation in standardised prices was also observed within each country. However, the variation would likely have been even larger if actual amounts on receipts had been directly used. In SE, prices for emergency procedures for dogs differed substantially between clinics, with the highest price being up to five times higher than the lowest. Median standardised prices for regular-hours caesarean section/ pyometra in SE were approximately €2,300, rising to nearly €4,000 for after-hours procedures in the final extraction.

The original version of this article has been updated.

