# Supplementary material for: Prices for veterinary care of dogs, cats and horses in selected countries in Europe
Source: Front Vet Sci. 2024 Jul 18;11:1403483. doi: 10.3389/fvets.2024.1403483 (PMC11292583; doi:10.3389/fvets.2024.1403483)
Supplement: Supplementary file 2 [file Data_Sheet_2.docx]

***Supplementary Material***

**Prices for veterinary care of dogs, cats and horses in selected countries in Europe**

**Agneta Egenvall*, Odd Höglund, Ruben Hoffman, Paul S Valle, Pia Haubro Andersen, Cecilia Lönnell, Anna Byström, Brenda N Bonnett**

**Correspondence:** Agneta Egenvall: agneta.egenvall@slu.se

Searches for veterinary clinics and practicing veterinarians were made in online business directories covering SE, NO, DK, UK and IR. The URLs used for data retrieval are found below. In case the search results were returned across multiple pages, repeat requests were made to ensure that all search results were retrieved from each search. The URLs used were used in order to cover most clinics in SE, NO and DK, and a sample for UK and IR (the URL used for the UK only covered only part of the available clinics).

The information retained from the searches was the name of the veterinary clinic, including all business operators providing veterinary care to animals i.e. from animal hospitals to single ambulatory veterinarians. Depending on the information available, the postal address, country, phone number, geo-coordinates, organization number, opening hours and homepage was retrieved. The results from each search were then cross-matched based on homepage URLs, phone numbers, and geolocation to merge search results for the same clinic across different sources. This information was then stored in a database using sqlite3 ([1](https://www.sqlite.org/about.html)). If more than one homepage URL was found for a clinic, all available URLs were stored in the database.

Each URL was requested using libcurl/C++ Requests (2) and links were extracted from the landing page as well as from all pages linked to on the landing page (ignoring external links). These links were then filtered using the following regular expression (https://en.wikipedia.org/wiki/Regular_expression): ‘(pris|pric)’. Links matching this expression were requested and page contents were searched for additional links, that is, pages with a URL that matched the filter were searched at an additional level of depth (all links on a homepage that matched the filter were searched and all links found that matched the filter was progressively added to the search strategy), to ensure that all links to pages with price information were retrieved.

Data were included over a period of 15 months because the first extraction at the VP-site was not complete for all procedures (i.e. for some procedures not all posts were shown, see further below for specifics of how this was handled). Thus, the web search was initiated in autumn 2022 and was repeated five times with approximate tri-monthly intervals. Web extractions were made 2022-08-30, 2022-09-26 (I), 2023-01-02 II), 2023-04-07, 2023-04-13 (III), 2023-06-29 IV), 2023-09-30 (V) and 2024-01-02 (VI). Web and VP-extractions were intended to be simultaneous, but for technical reasons the first extraction from VP was done in 2022-08-30 and the third 2023-04-13. Note that it would occasionally occur that a clinic’s homepage was available at one point in time but not another, which could be due to change of homepage address, server temporary unavailable, technical failure etc.

The results from the sql-database were downloaded to an excel file that contained 337,583 rows for all 6 extractions.

Links:

Eniro. (SE). https://www.eniro.se/veterin%C3%A4r/f%C3%B6retag (SE) [accessed September 15, 2022]

Anicura. (SE, NO, DK). https://www.anicura.se/vara-djursjukhus-och-kliniker/ [accessed September 15, 2022]

Golden pages (IR). https://www.goldenpages.ie/q/business/advanced/what/Vets/ (IR) [accessed September 15, 2022]

Find Any UK Vet. (UK). https://www.any-uk-vet.co.uk (UK) [accessed September 15, 2022]

Gule sider. (NO). https://www.gulesider.no/api/cs?query=veterin%C3%A6r&profile=no (NO) [accessed September 15, 2022]

Krak. (DK). https://www.krak.dk/api/cs?query=veterin%C3%A6r&profile=krak [accessed September 15, 2022]

Evidensia djursjukvård. (SE). https://evidensia.se/klinik (SE) [accessed September 15, 2022]

Vettris veterinärklinik. https://www.vettris.se/ (SE) [accessed September 15, 2022]

Vetpris. https://www.vetpris.se/ (SE, NO, DK - clinic and price information only, clinic URLs not available) [accessed September 15, 2022]

References:

1. SQlite (version 3.39.2) [https://www.sqlite.org](https://www.sqlite.org/about.html) [accessed July 21, 2022].

2. GitHub. (GIT_TAG 7a02d679308a19220e3b75616c18ff9e8deb5f9a, version 1.9.1 July 31, 2022). <https://github.com/libcpr/cpr/commit/7a02d679308a19220e3b75616c18ff9e8deb5f9a>

[accessed October 20, 2022].
