# Supplementary material for: Prices for veterinary care of dogs, cats and horses in selected countries in Europe
Source: Front Vet Sci. 2024 Jul 18;11:1403483. doi: 10.3389/fvets.2024.1403483 (PMC11292583; doi:10.3389/fvets.2024.1403483)
Supplement: Supplementary file 3 [file Image_1.pdf]

Supplemental Figure 1. PPP exchange adjusted currencies and currencies in Euro for gonadectomy in cats and dogs using data from Tables 2 and 3. Prices extracted from the web of the veterinary clinics (web) and the price-comparison site (vetpris.se, VP, (the VP price is originally web-based)) at 6 extractions (Time), approximately every three months, during autumn 2022 – winter 2023/2024. Currencies are shown by country (Norway (NO), Sweden (SE), UK and Denmark (DK), source (direct web or via VP, and extraction (Time I-VI). The PPP-adjusted currencies are adjusted using data from https://data.oecd.org/conversion/purchasing-power-parities-ppp.htm, from year 2022.

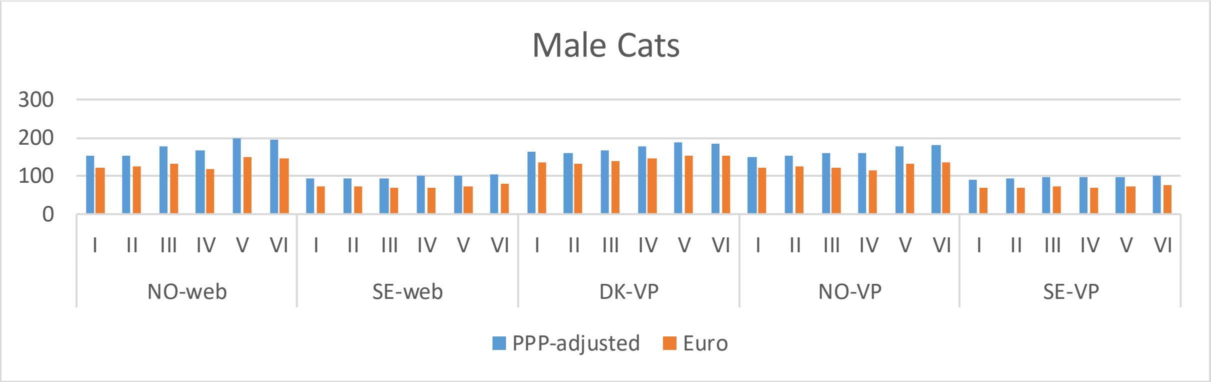
Male Cats
300
200
100
0
I II III IV V VI
NO-web SE-web DK-VP NO-VP SE-VP
PPP-adjusted Euro

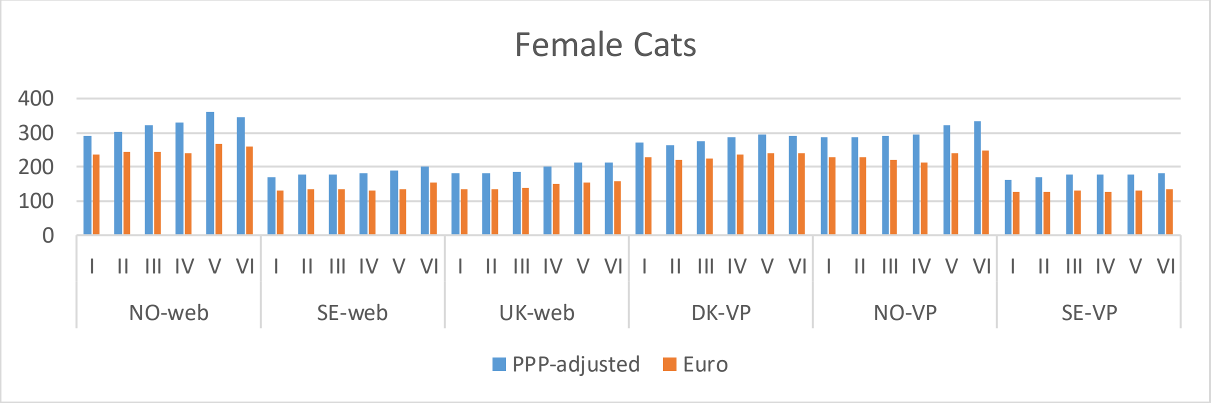
Female Cats
400
300
200
100
0
I II III IV V VI
NO-web SE-web UK-web DK-VP NO-VP SE-VP
PPP-adjusted Euro

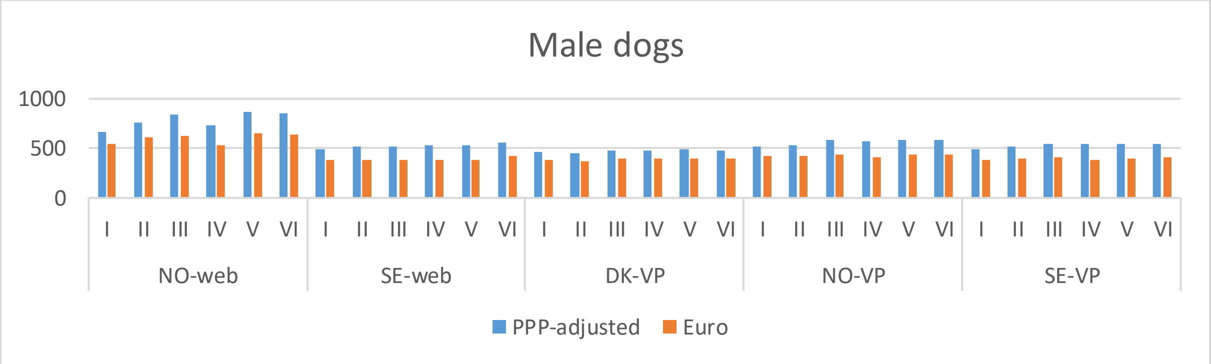
Male dogs
1000
500
0
I II III IV V VI
NO-web SE-web DK-VP NO-VP SE-VP
PPP-adjusted Euro

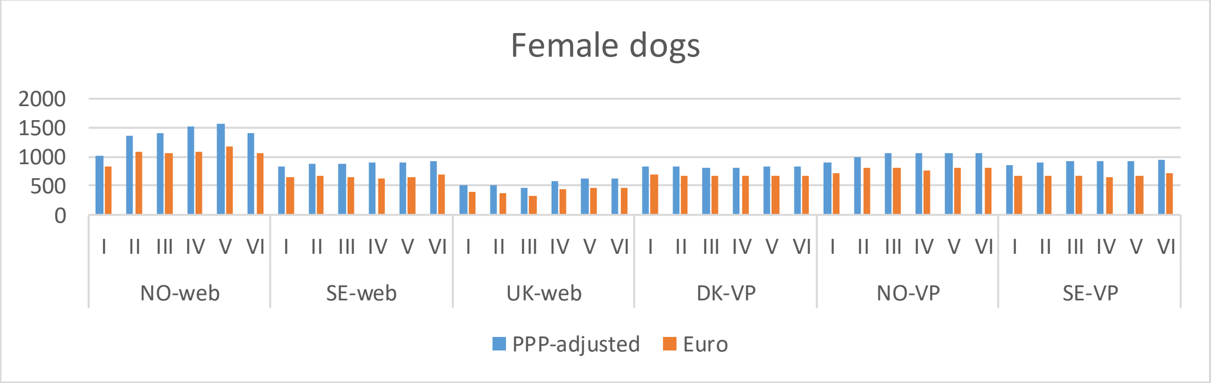
Female dogs
2000
1500
1000
500
0
I II III IV V VI
NO-web SE-web UK-web DK-VP NO-VP SE-VP
PPP-adjusted Euro
